# Phenotypic variation in otolith shape of American shad across eastern North American rivers

**DOI:** 10.1038/s41598-025-14742-1

**Published:** 2025-08-13

**Authors:** Joana Vasconcelos, Karin E. Limburg, José Luis Oterro-Ferrer, Víctor M. Tuset

**Affiliations:** 1 Grupo en Biodiversidad y Conservación (BIOCON), ECOAQUA, Universidad de Las Palmas de Gran Canaria, Canary Islands, 35017 Spain; 2 MARE - Marine and Environmental Sciences Centre/ARNET - Aquatic Research Network, Agência Regional para o Desenvolvimento da Investigação Tecnologia e Inovação (ARDITI), Funchal, Portugal; 3 Department of Aquatic Resources, Swedish University of Agricultural Science, Almas Allé 5, Box 7018, 75007 Uppsala, Sweden; 4 SUNY College of Environmental Science and Forestry, Syracuse, NY 13210 USA; 5 Biostatech, Advice, Training and Innovation in Biostatistics (Ltd), Santiago de Compostela, Spain; 6 Instituto de Oceanografía y Cambio Global, IOCAG, Universidad de Las Palmas de Gran Canaria, Unidad Asociada ULPGC-CSIC, Canary Islands, Spain

**Keywords:** Animal migration, Ichthyology, Freshwater ecology

## Abstract

Otolith shape analysis has been widely applied to study population structure and environmental influences in various fish species. However, research on American shad (*Alosa sapidissima*) otolith morphology remains scarce, despite its potential to provide insights into population differentiation and environmental adaptation. This study analyses otolith contour shape from 1141 American shad collected between 2000 and 2023 across eleven large rivers from Canada to Florida. Using a wavelet transform framework based on the à trous algorithm and B3-spline wavelet, we quantified otolith shape variability and assessed its effectiveness for population discrimination. Principal Component Analysis revealed significant shape variation, with key differences in the *rostrum*, *antirostrum*, and posterior region. Wavelet analysis identified two primary otolith morphologies—upper and lower *rostrum*—geographically structured along a latitudinal gradient. A Multilayer Perceptron neural network successfully classified individuals with 90.9% accuracy, highlighting strong population differentiation, particularly in the St. Lawrence and Delaware rivers. Cluster analysis identified five morphotypes with distinct spatial distributions, suggesting a role for local environmental conditions in shaping otolith morphology. These findings underscore the utility of otolith shape analysis in deciphering population structure and highlight potential links between environmental variation and phenotypic plasticity in American shad.

## Introduction

Population connectivity is a critical component of anadromous fish life history, influencing the persistence of local populations as these species rely on both freshwater and marine habitats at different stages of their life cycle^1,2^. In North America, the American shad (*Alosa sapidissima*) is an anadromous species whose connectivity has been greatly altered by human activities. American shad have a broad endemic distribution, extending from the St. Lawrence River and the Canadian Maritimes to the St. Johns River in Florida^3^, a range of about 20 latitudinal degrees. American shad spawn mainly in large rivers and larger tributaries. However, habitat modifications, including dam construction, channel dredging, and shoreline hardening, have significantly reduced available shad spawning habitats^3^. Recent analysis estimates that damming has led to a one-third reduction in American shad populations, from more than 69 million spawners to less than 42 million^4^. Additionally, rising ocean temperatures are associated with decreased growth of shad^5^, with projections suggesting further declines and increased mortality due to climate change. Notably, these studies indicate that modeling shad populations at the individual river level provides a better fit than regional-scale models and highlights the role of local environmental factors in shaping adaptations.

Otoliths serve as valuable tools for reconstructing life history traits, providing insights into age, growth, migration patterns, and other ecological aspects^6–10^. Given their ability to encode environmental and biological information, otoliths have been widely used to investigate population structure and habitat use in many fish species, including American shad (e.g.,^11–14^).

There has been extensive work using American shad otoliths for age determination^14–16^, otolith microstructure, and trace element composition for natal river identification and migratory patterns^11–13,17^. However, studies on the morphological characteristics of their otoliths remain scarce. With the exception of a master’s thesis on hickory shad (*Alosa mediocris*) that explored otolith shape for population identification^18^, little attention has been given to how otolith shape varies within and among North American alosid populations.

This knowledge gap presents an opportunity to investigate whether otolith shape reflects population structuring, environmental adaptation, or both. To address this, we employ wavelet transform analysis, a method that enhances the ability to characterize otolith contour variation at multiple spatial scales. Advances in digital signal processing have introduced robust alternatives to traditional Elliptic Fourier descriptors for otolith shape analysis. The wavelet approach, specifically the à trous algorithm paired with a B3-spline wavelet, developed by the AFORO team (http://aforo.cmima.csic.es/), enables a multiscale evaluation of otolith contours^19,20^, capturing fine-scale structural nuances while preserving overall shape integrity. This method has proven useful in studies of interspecific diversity^21^, intraspecific phenotypic variability^22,23^ and stock delineation^24,25^. Given its precision, wavelet analysis holds promise for tracing natal origins, detecting natal homing behaviors and refining our understanding of population connectivity in American shad.

We hypothesize that habitat variability along the eastern North American coasts shapes the otolith morphology of American shad, driving population differentiation through local environmental adaptations. Building on Vignon’s^26^ demonstration of how habitat heterogeneity shapes otolith development, this study aims to (i) evaluate the effectiveness of wavelet analysis for American shad otolith contour shape analysis, (ii) identify the specific otolith zones contributing to population discrimination across rivers, and (iii) assess the degree of phenotypic differentiation when treating all individuals as a metapopulation, offering new insights into population structure and environmental plasticity.

## Methods

### Data collection

**Fig. 1:**
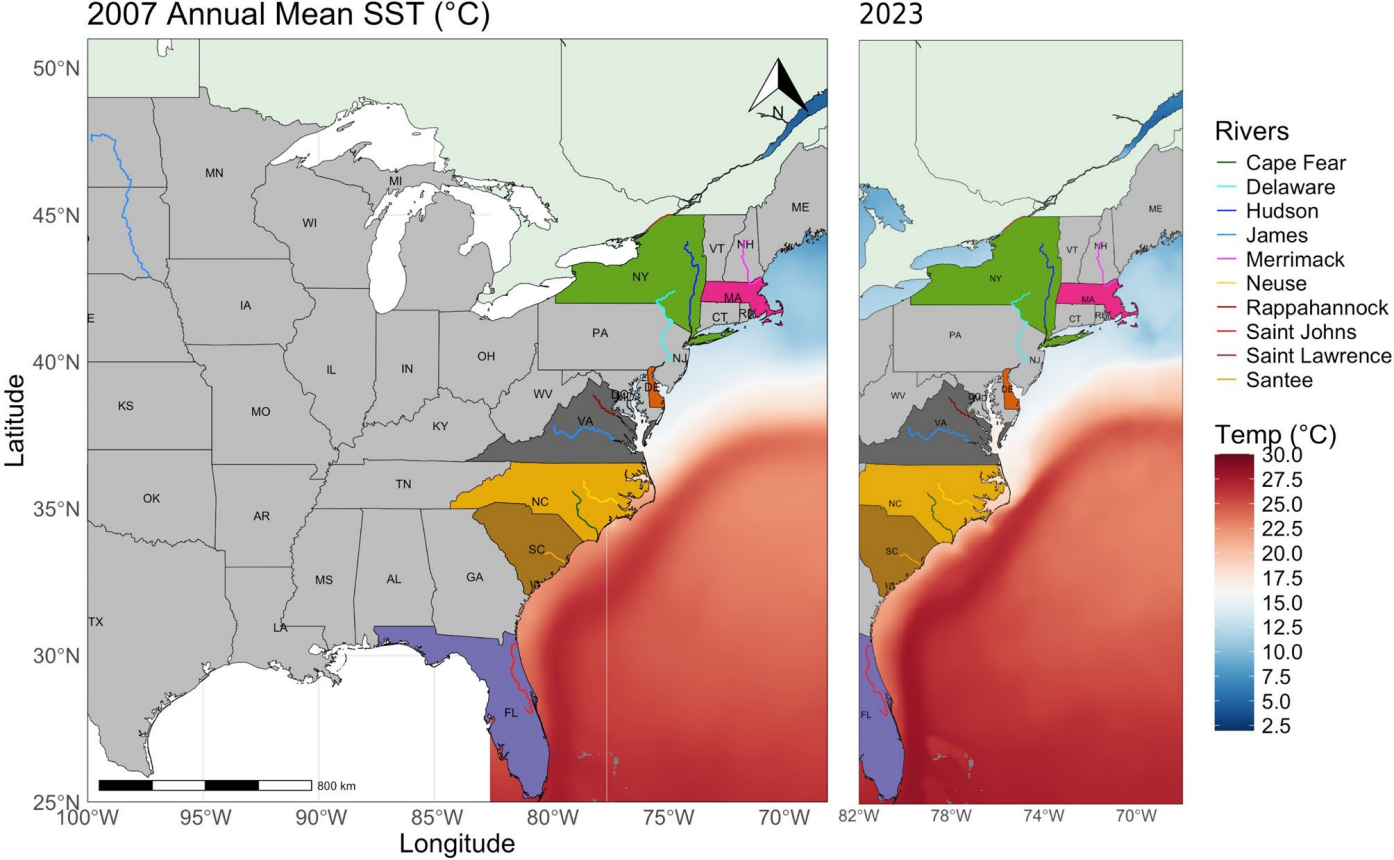
Map showing the collection sites of *A. sapidissima*, from the St. Lawrence River in Quebec to the St. Johns River in Florida, including the Merrimack (MA), Hudson (NY), Delaware (DE), Rappahannock (VA), York (VA), James (VA), Neuse (NC), Cape Fear (NC), and Santee (SC) rivers. The maps also show the annual mean sea surface temperatures (SST, $^{\circ }$C) for the years 2007 and 2023, representing the temporal endpoints of the study period. SST data were obtained from the OSTIA dataset (^27^; https://doi.org/10.48670/moi-00168).

This study analysed 1,141 *A. sapidissima* specimens collected between 2005 and 2023 from eleven rivers across one province in Canada and seven U.S. states (Fig. 1). Sampling locations included the St. Lawrence River (STL; 2014 and 2019) in Quebec (QC), the Merrimack River (MER; 2012 and 2022) in Massachusetts (MA), the Hudson River (HUD; 2018 and 2021) in New York (NY), the Delaware River (DEL; 2005–2007) in Delaware (DE), the Rappahannock (RAPP; 2020), York (YOR; 2018 and 2021) and James (JAM; 2020) rivers in Virginia (VA), Neuse (NEU; 2021) and Cape Fear rivers (CF; 2023) in North Carolina (NC), the Santee River (SAN; 2021) in South Carolina (SC), and the St. Johns River (STJ; 2022-2023) in Florida (FL) (Table 1). Global SST data were obtained from the OSTIA dataset (^27^; https://doi.org/10.48670/moi-00168) for the period 2007–2023. Data for 2005 and 2006 were not available for the sampling areas.

**Table 1:** Number of individuals (N), mean total length (TL, mm), and otolith length (OL, mm), with standard deviations (SD), for *A. sapidissima* sampled from eleven rivers along the North American East Coast, ordered from north to south, from the St. Lawrence River in Quebec to the St. Johns River in Florida. River codes and states are provided in the table.

State/province	River code	Population	N	TL		OL	
				Mean ± SD	Min.–Max.	Mean ± SD	Min.–Max
QC	STL	St. Lawrence River	85	520 ± 32	455–598	4.29 ± 0.24	3.60–4.87
MA	MER	Merrimack River	278	468 ± 35	379–579	4.01 ± 0.26	3.36–4.83
NY	HUD	Hudson River	49	494 ± 32	409–572	4.19 ± 0.28	3.60–4.72
DE	DEL	Delaware River	26	498 ± 36	440–558	4.09 ± 0.29	3.45–4.63
VA	RAPP	Rappahannock River	86	500 ± 18	441–538	4.09 ± 0.23	3.27–4.54
VA	YOR	York River	78	496 ± 27	439–556	4.12 ± 0.24	3.64–4.62
VA	JAM	James River	18	495 ± 22	451–536	4.10 ± 0.24	3.74–4.53
NC	NEU	Neuse River	89	464 ± 39	382–541	4.15 ± 0.26	3.39–4.73
NC	CF	Cape Fear River	120	447 ± 38	348–536	3.98 ± 0.26	3.52–4.93
SC	SAN	Santee River	77	460 ± 40	386–563	4.15 ± 0.29	3.61–4.81
FL	STJ	St. John’s River	235	426 ± 32	345–496	3.91 ± 0.22	3.83–4.66

### Contour shape analysis

**Fig. 2:**
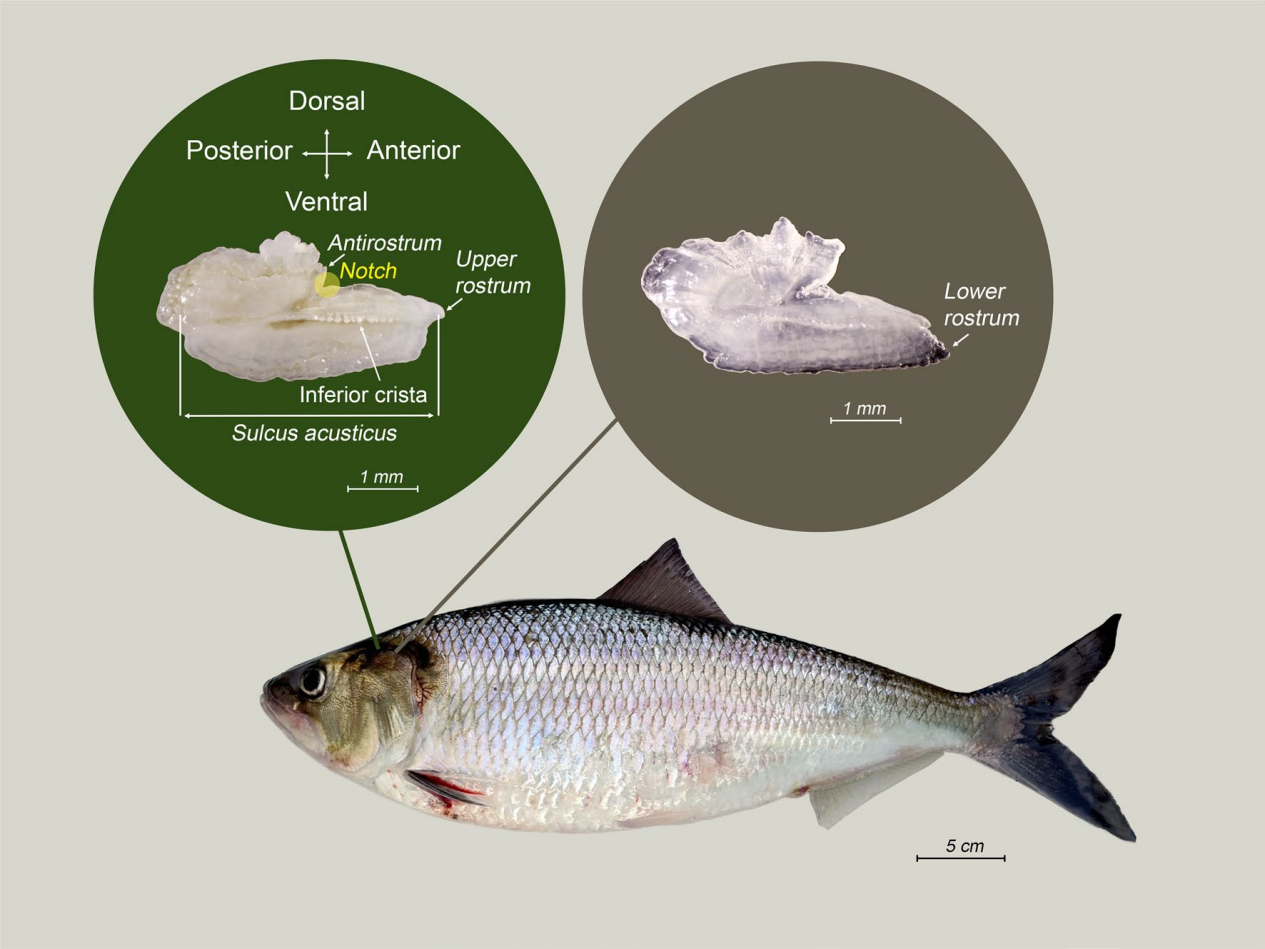
Representation of an American shad measuring 52 cm in total length, highlighting the otolith’s *rostrum* morphometry. The left inset illustrates the upper *rostrum*, showing key anatomical features such as the *antirostrum*, *notch*, and *sulcus acusticus*. The right inset presents the lower *rostrum*.

Left otoliths were placed on a dark background with the *sulcus acusticus* facing up and the *rostrum* to the right (Fig. 2). They were photographed using an AmScope USB Microscope camera MU500 (5 megapixels) connected to a SZ-40 Olympus stereomicroscope and measured to 0.01 mm using ImageJ software^28^. For contour shape analysis, 512 equidistant Cartesian coordinates were extracted from each otolith’s orthogonal projection using a wavelet transform^25,29^. We focused on the 4^th^ of the nine wavelets generated, as it has been shown to be the most effective for population discrimination^21,25^, capturing subtle phenotypic differences within species^19,22,23^.

### Statistical analysis

We followed the methodology outlined by the AFORO team (see^19,20^). To reduce the dimensionality of the wavelet functions data set while retaining most of the information, we performed a Principal Component Analysis (PCA) using the variance-covariance matrix. Principal components were selected using the broken-stick model, keeping only those with eigenvalues explaining more variance than expected by chance^30^. To address potential allometric effects on intraspecific variation, Pearson’s correlations were calculated between otolith size and the principal components. Any influence of otolith size was mitigated by constructing a new PCA matrix based on the residuals from common within-group slopes of linear regressions between each component and otolith size^31^. Total variation among locations was then assessed using permutational multivariate analysis of variance (PERMANOVA;^32^), applying 9,999 permutations with Manhattan distance, and Bonferroni corrections for post-hoc pairwise comparisons. For population classification, we employed the Multilayer Perceptron (MLP)^23,33^, a powerful neural network model. To address unbalanced datasets, we integrated the Synthetic Minority Oversampling Technique (SMOTE)^34^, enhancing the model’s ability to learn from underrepresented groups. Given the small sample size, we used Leave-One-Out Cross-Validation (LOOCV)^35^, with each observation serving as the validation set and the remaining data used for training. The model applied pre-processing to the predictor data using the ’center’ and ’scale’ methods, standardizing all predictors—a critical step for machine learning models like MLP, which are sensitive to variations in scale. We identified optimal hyperparameters, including the number of hidden units (i.e., neurons in the hidden layer of the MLP model), through preliminary tuning. To ensure robustness, the validation process was repeated 1,000 times for each analysis. Cross-tabulations of observed and predicted classes, along with key metrics, such as overall accuracy and Cohen’s kappa, were generated. The classifications were performed using the R packages *caret* v.6.0.94^36^ and *RSNNS* v.0.4.16^37^. To identify morphotypes within the entire sample, hierarchical clustering with “ward.D” agglomeration and the *cutree()* function segmented the dendrogram tree^38^. All analyses were performed in R (v4.4.1;^39^).

## Results

### Population ID

**Fig. 3:**
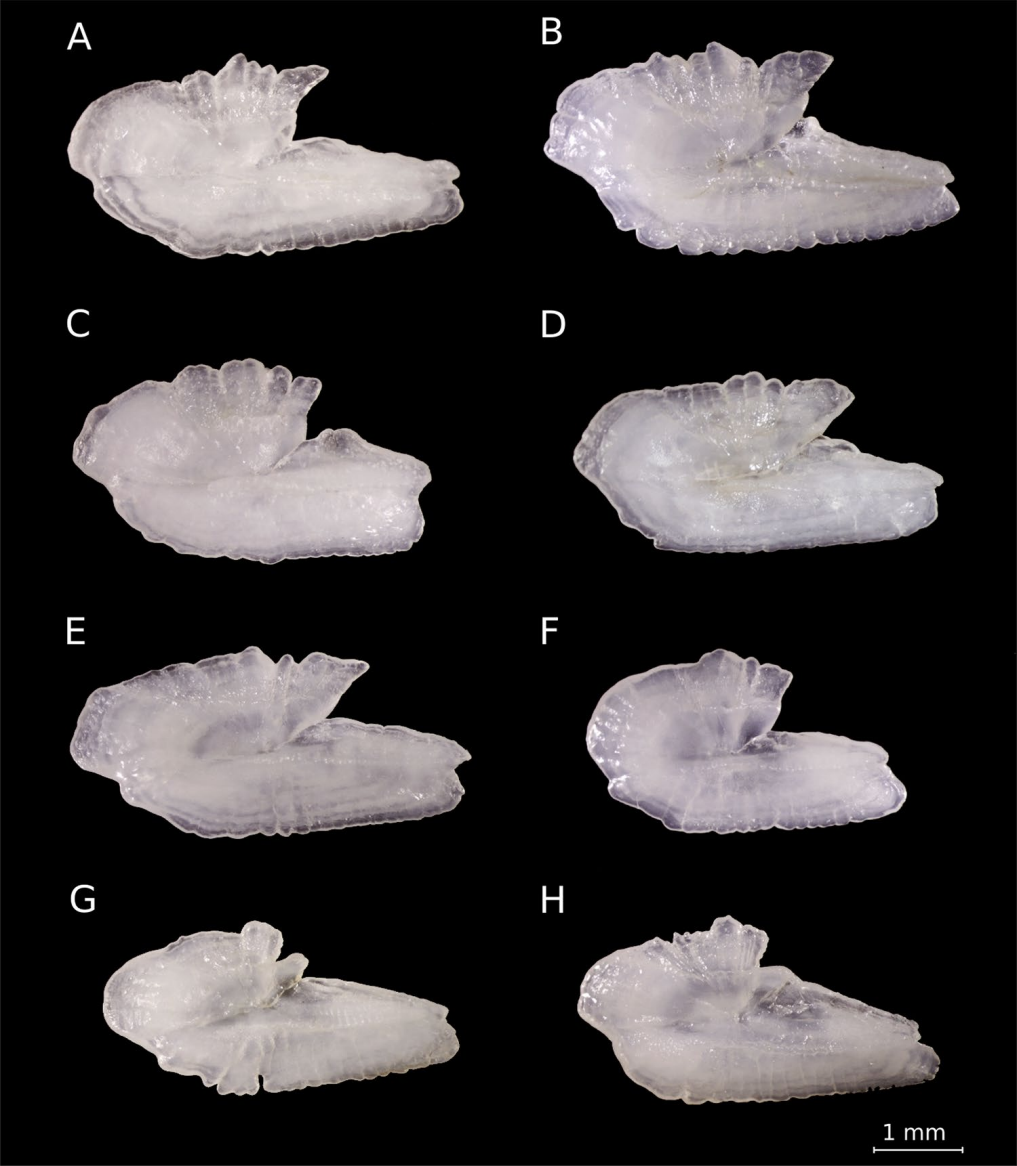
American shad otoliths. Examples are shown from the St. Lawrence River (**A**), Hudson River (**B**), Merrimack River (**C**,**D**), Rappahannock River (**E**), Cape Fear River (**F**), and St. Johns River (**G**,**H**).

**Fig. 4:**
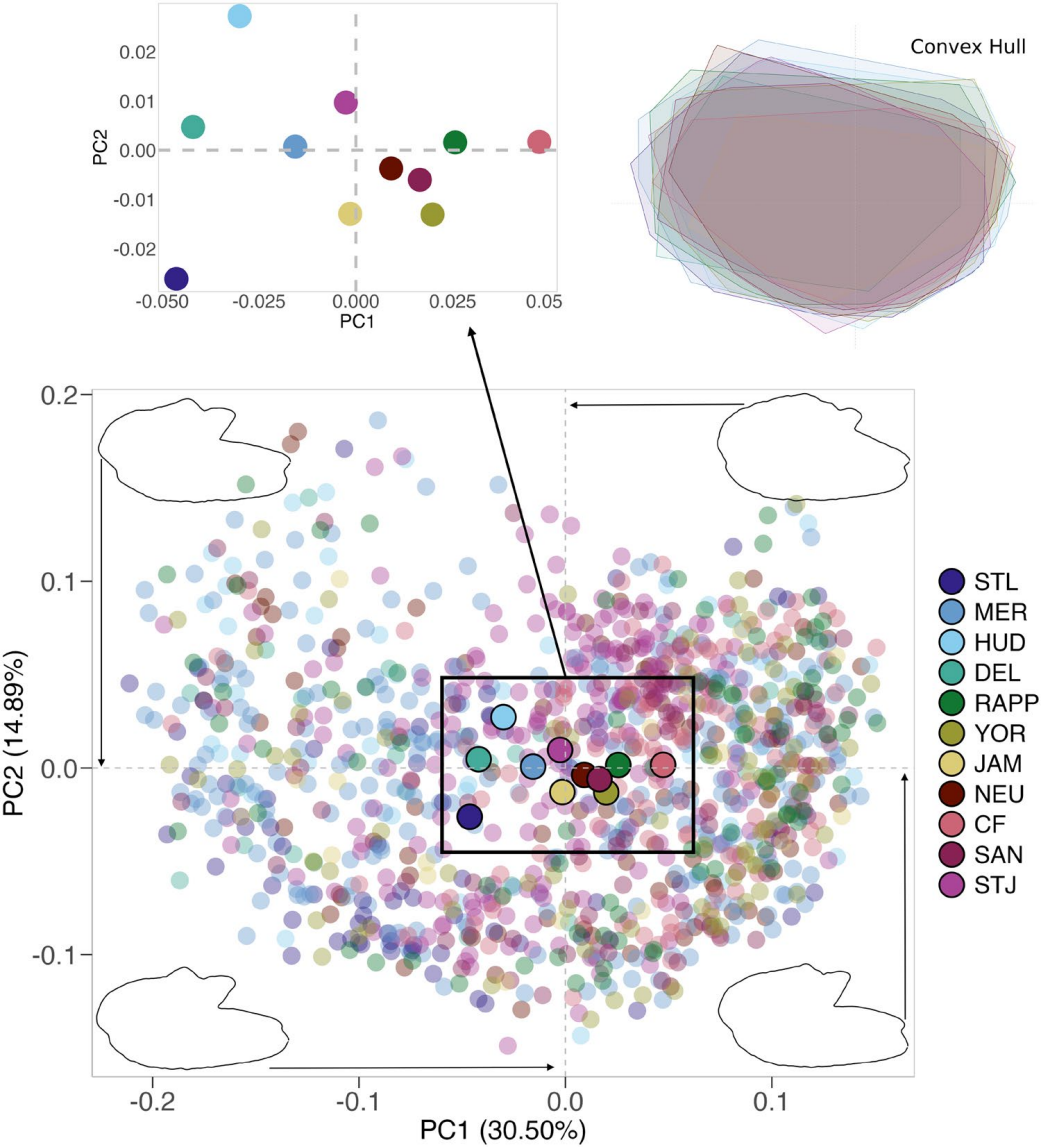
Scatterplot of the principal components of *A. sapidissima* from eleven populations sampled across Quebec (QC) to Florida (FL). Sampling locations include the St. Lawrence (STL), Merrimack (MER), Hudson (HUD), Delaware (DEL), Rappahannock (RAPP), York (YOR), James (JAM), Neuse (NEU), Cape Fear (CF), Santee (SAN), and St. Johns (STJ) rivers. Convex hulls, represented as polygons, enclose the outermost data points for each sampling location. Shape contours corresponding to extreme PC1 values illustrate otoliths with a pronounced upper *rostrum* (minimum negative PC1) and a lower *rostrum* (maximum positive PC1). Similarly, extreme PC2 values highlight otoliths with a more pronounced (minimum PC2) and less pronounced (maximum PC2) *antirostrum*.

The otoliths of American shad exhibited pronounced shape variability within rivers (Fig. 3). Differences in the *rostrum* and *antirostrum* sizes, *excisura ostii* width, *notch* depth, and posterior region strongly shaped their overall structure. This diversity resulted in an extensive spread in PCA morphospace (PC1 vs. PC2, Fig. 4). Principal Component Analysis (PCA) revealed that the first 20 components captured 92.6% of the total variance, indicating that variance is spread across multiple dimensions. The first principal component (PC1), accounting for 30.9% of the variance, differentiated otoliths based on the relative position of the opening of the inferior crista in the *ostium* to the farthest point from the centroid. Negative PC1 scores corresponded to otoliths with an inferior crista ending below this point, referred to as the upper *rostrum*, and a more angled posterior, while positive scores indicated an ending above this point, or lower *rostrum* (Fig. 4).

The second principal component (PC2), representing 14.9% of the variance, distinguished otoliths with a prominent *antirostrum* and adjacent region and a deeper *notch* (negative PC2 scores) from those with wider shapes, a less developed *antirostrum*, and a shallower *notch* (positive PC2 scores) (Fig. 4). Despite considerable morphological heterogeneity within each population (illustrated by the convex hull in Fig. 4), some geographic separation is apparent, with northern populations clustering at negative values and southern populations at positive values. The PERMANOVA analysis indicated clear population differentiation (F = 5.04,* P* = 0.001), though with post-hoc pairwise comparisons revealing no significant differences across all populations (Table S1).

**Fig. 5:**
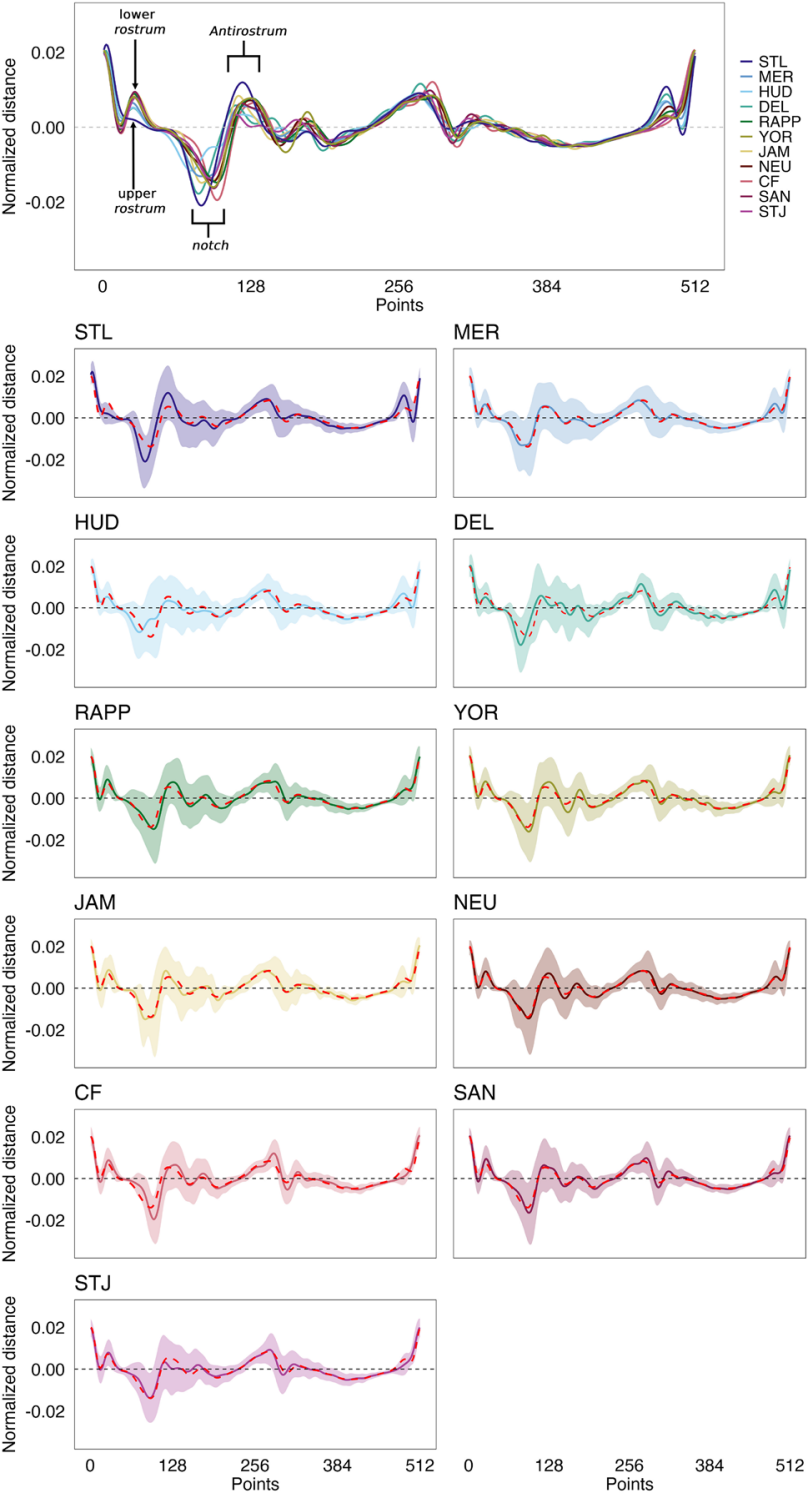
Mean (solid line) and standard deviation (shading) of otolith contour decomposition of *A. sapidissima* from eleven populations sampled across Quebec (QC) to Florida (FL). Sampling locations include the St. Lawrence (STL), Merrimack (MER), Hudson (HUD), Delaware (DEL), Rappahannock (RAPP), York (YOR), James (JAM), Neuse (NEU), Cape Fear (CF), Santee (SAN), and St. Johns (STJ) rivers. The dashed line represents the overall mean otolith contour decomposition for all populations combined. The X-axis represents 512 equidistant points along the otolith perimeter, while the Y-axis represents the mean normalized distance. The 4^th^ of the nine wavelets was selected for its effectiveness in distinguishing populations.

Wavelet analysis (Fig. 5) and contour plots (Fig. S1) revealed that regional variability was mainly driven by differences in the shape and size of the *rostrum* and *antirostrum*, including the *notch*; variations in otolith width, which influenced its overall structure, and features in the posterior region were also important. However, the primary distinguishing feature among rivers is the position of the inferior crista’s opening, which either ends below or above the farthest point from the centroid to the otolith perimeter. This distinction defines two geographically structured clusters: (a) a northern cluster, where the inferior crista terminates below the farthest point from the otolith centroid (upper *rostrum*; Fig. 2), including populations of the St. Lawrence, Merrimack, Hudson, and Delaware rivers. Within this group, the St. Lawrence otoliths exhibit a unique overall contour shape with a deeper *notch* contrasting with the shallower one of Hudson population (Fig. S1), while Delaware shares some characteristics with the southern group, such as the posterior margin; (b) a southern cluster, where the inferior crista ends above the farthest point from the centroid (lower *rostrum*; Fig. 2), includes populations from Rappahannock, York, James, Neuse, Cape Fear, Santee and St. Johns. These southern populations are further characterized by wider otoliths that expand beyond the *antirostrum* toward the posterior, with some exhibiting a sharper angle along the posterior margin. Like the St. Lawrence in the northern group, the St. Johns River population, at the southern end of the species range, represents a morphological extreme within the southern population.

**Fig. 6:**
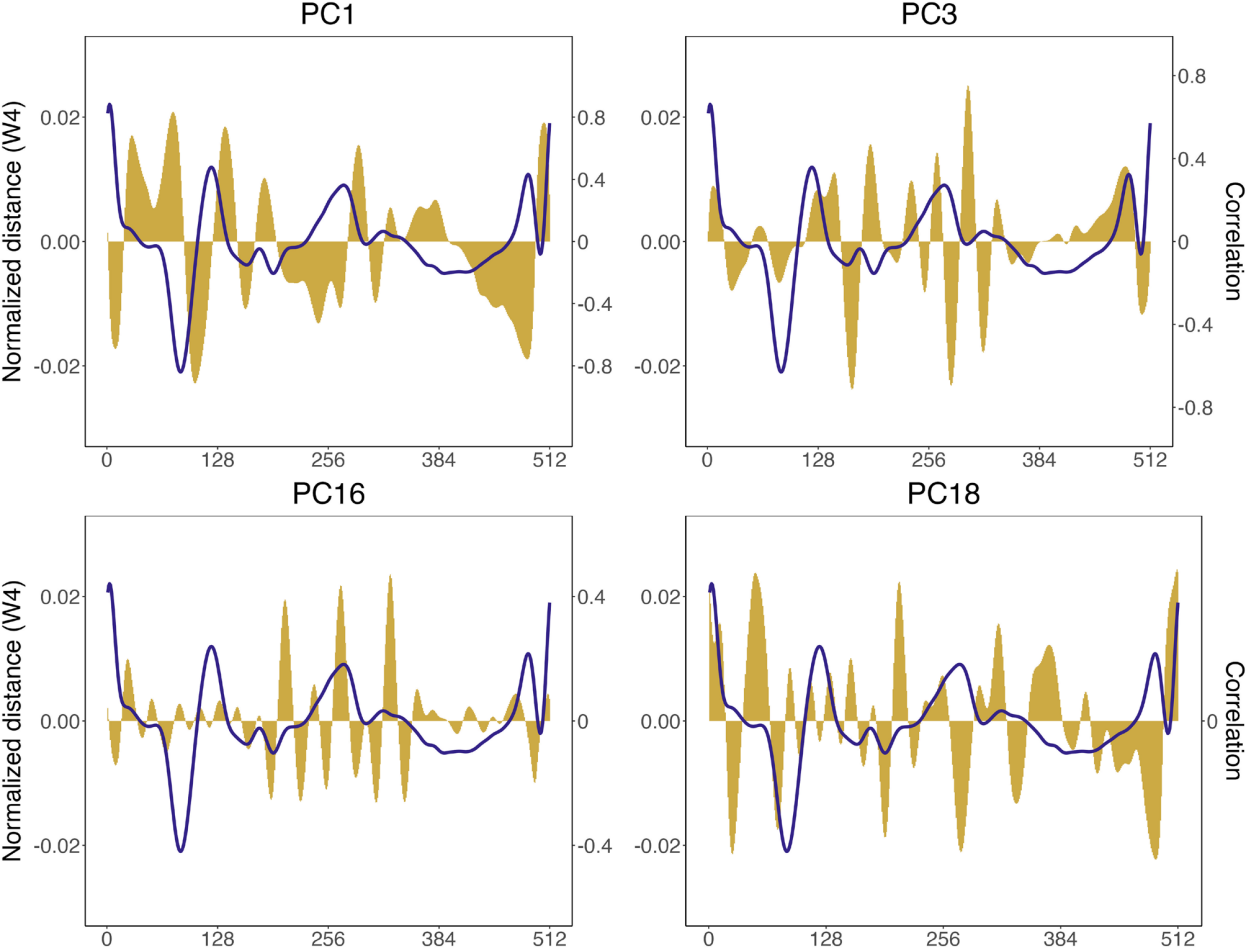
Correlations (yellow) of the most relevant principal components (PCs) for classification with the normalized distance of the 4^th^ wavelet for *A. sapidissima*, sampled from eleven populations along the North American East Coast, from the St. Lawrence River in Quebec to the St. Johns River in Florida. The 4^th^ wavelet, shown as an example (solid line), represents the St. Lawrence River population and was selected for its effectiveness in distinguishing among populations.

The overall classification accuracy was 90.9%, with a Cohen’s kappa of 0.895. Accuracy varied across populations, ranging from 82.1% in the STJ population to 100% in the Delaware, York and James populations (Table 2). The key principle components driving this classification included PC1, which was influenced by the morphology and size of the *rostrum* and *antirostrum*, as well as the *notch* depth, PC3, correlated with the posteroventral margin, PC16, associated with the dorsal and postero-ventral margins, and PC18, reflecting variations mainly in the anterior margin (Fig. 6).

**Table 2:** Confusion matrix (LOOCV) showing phenotype abundances, classification accuracy, and Cohen’s kappa index (k) for *A. sapidissima*, classified using the MLP classifier. Data includes eleven sampled populations, spanning Quebec (QC) to Florida (FL): St. Lawrence (STL), Merrimack (MER), Hudson (HUD), Delaware (DEL), Rappahannock (RAPP), York (YOR), James (JAM), Neuse (NEU), Cape Fear (CF), Santee (SAN), and St. Johns (STJ) rivers. Correct group classifications are highlighted in bold.

Prediction	References											Performance measures		
	STL	MER	HUD	DEL	RAPP	YOR	JAM	NEU	CF	SAN	STJ	Accuracy	k	%Accuracy
STL	**72**	1	0	0	0	0	0	0	1	1	2			84.71
MER	3	**257**	1	0	1	0	0	0	0	0	7			92.45
HUD	2	3	*46*	0	0	0	0	0	2	0	4			93.88
DEL	0	0	0	**26**	0	0	0	0	0	0	0			100
RAPP	3	1	1	0	**82**	0	0	0	1	2	4			95.35
YOR	1	2	0	0	0	**78**	0	0	1	1	3			100
JAM	0	0	0	0	0	0	**18**	0	1	1	4			100
NEU	1	5	0	0	2	0	0	**85**	0	0	9			95.51
CF	2	3	1	0	0	0	0	2	**109**	0	6			90.83
SAN	1	2	0	0	0	0	0	2	4	**71**	3			92.21
STJ	0	4	0	0	1	0	0	0	1	1	**193**			82.13
Total	85	278	49	26	86	78	18	89	120	77	235	0.9089	0.8945	

### Morphotypes identification

**Fig. 7:**
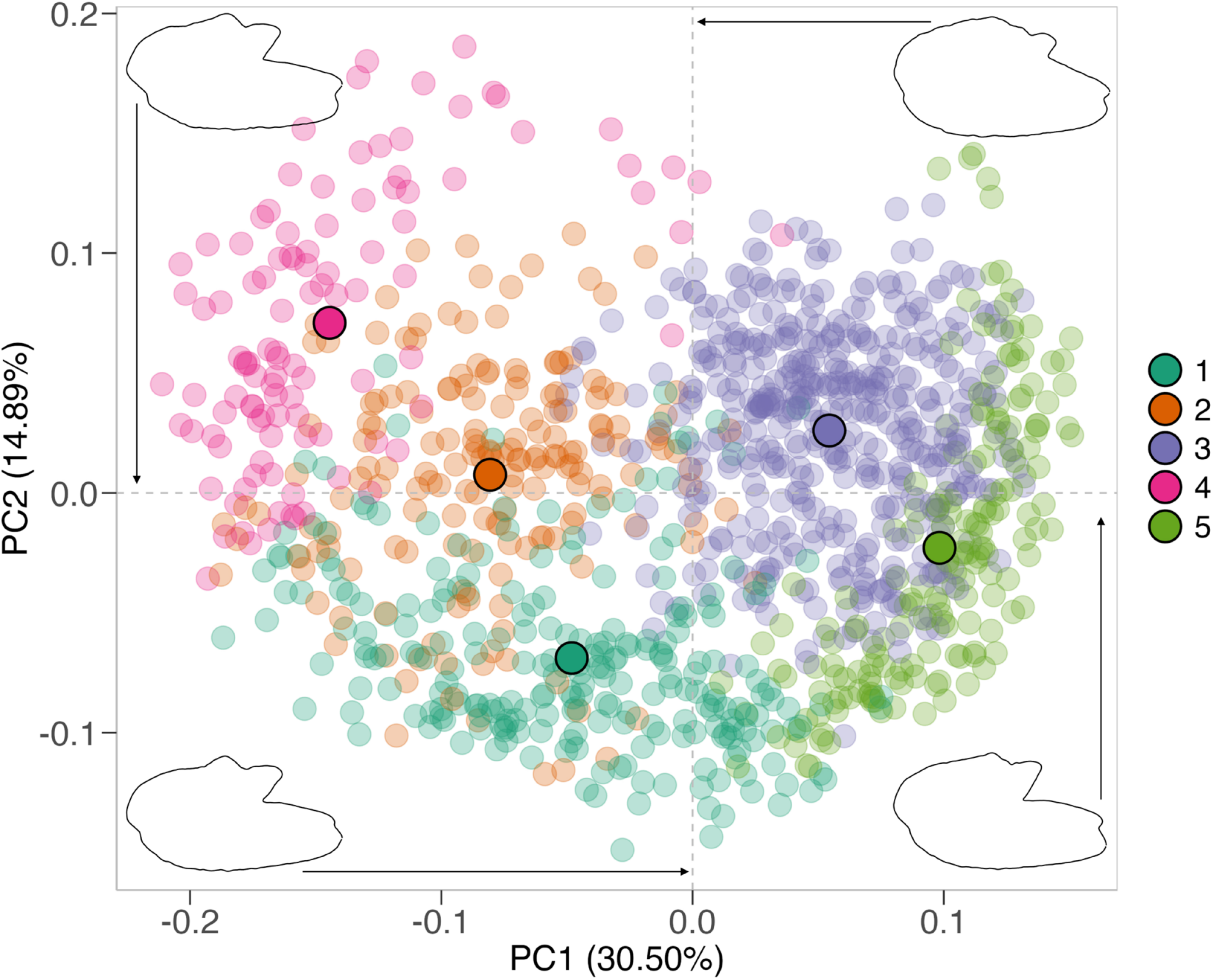
Scatterplots of principal component scores for the five morphotypes identified in *A. sapidissima*, sampled from eleven populations along the North American East Coast, from the St. Lawrence River in Quebec to the St. Johns River in Florida. Shape contours corresponding to extreme PC1 values illustrate otoliths with a pronounced upper *rostrum* (minimum PC1) and a lower *rostrum* (maximum PC1). Similarly, extreme PC2 values highlight otoliths with a more or less pronounced *antirostrum* (minimum vs. maximum PC2).

**Fig. 8:**
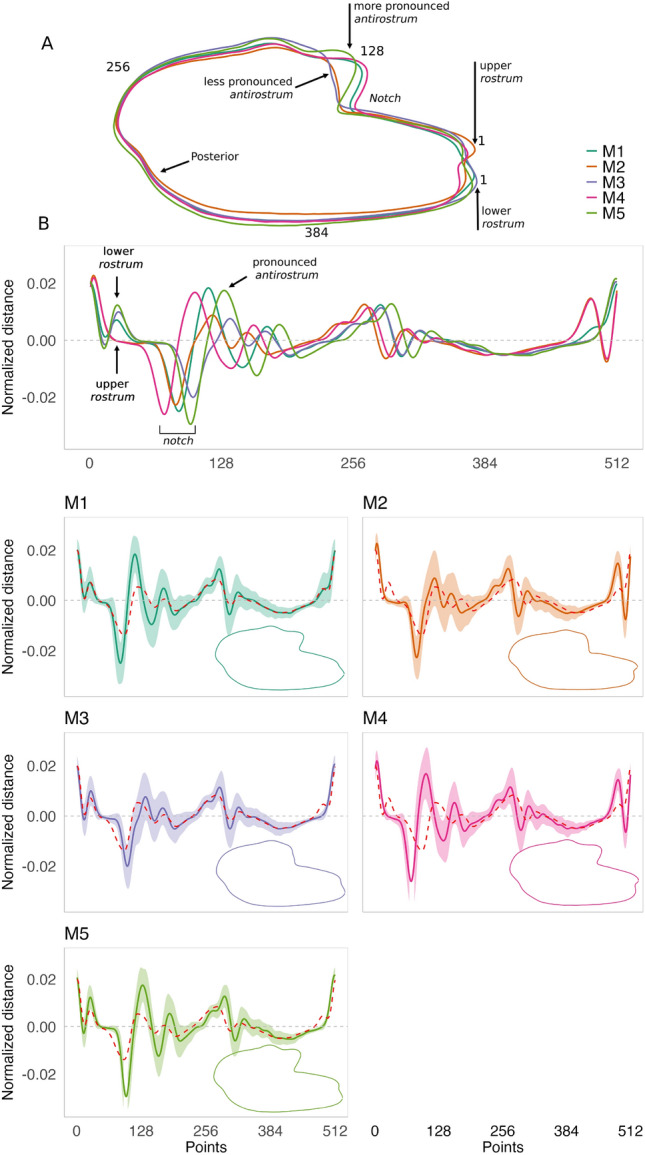
Average decomposition of otolith contours using the 4^th^ wavelet for the five phenotypes identified in *A. sapidissima*, sampled from eleven populations along the North American East Coast, from the St. Lawrence River in Quebec to the St. Johns River in Florida. The 4^th^ wavelet, selected for its effectiveness in distinguishing populations, is used for contour analysis. The dashed line represents the overall mean otolith contour decomposition across all populations. The first Cartesian coordinate is automatically determined by selecting the farthest point from the centroid to the otolith outline.

Using the “ward-D” agglomeration method, at least five morphotypes were identified (M1-5, Fig. S2), each distinctly positioned in morphospace (Fig. 7). The PC1 axis separated morphotypes based on the termination of the inferior crista: M2 and M4 (negative PC1 scores) had the crista ending below the farthest point from the centroid, while M3 and M5 (positive PC1 scores) had it ending above (Figs. 7 and  8). M2 and M4 differed further, with M4 displaying a wider otolith (positive PC2 scores) and a more pronounced *antirostrum*, compared to M2’s more elliptical shape and subtler *antirostrum*.

M3 and M5 were distinguished by M3’s less pronounced *antirostrum* and shallower *notch* (positive PC2 scores) versus M5’s deeper *notch* and more defined *antirostrum* (negative PC2 scores). Morphotype M1, characterized by intermediate features such as an elongated shape, a prominent *antirostrum*, a deeper *notch*, and a crista that ends at or slightly above the farthest point, was distinguished by negative PC2 scores (Figs. 7 and  8). PERMANOVA confirmed significant differences among morphotypes (F = 112.01,* P* = 0.001), with all pairwise comparisons showing significance (*P* = 0.01).

**Fig. 9:**
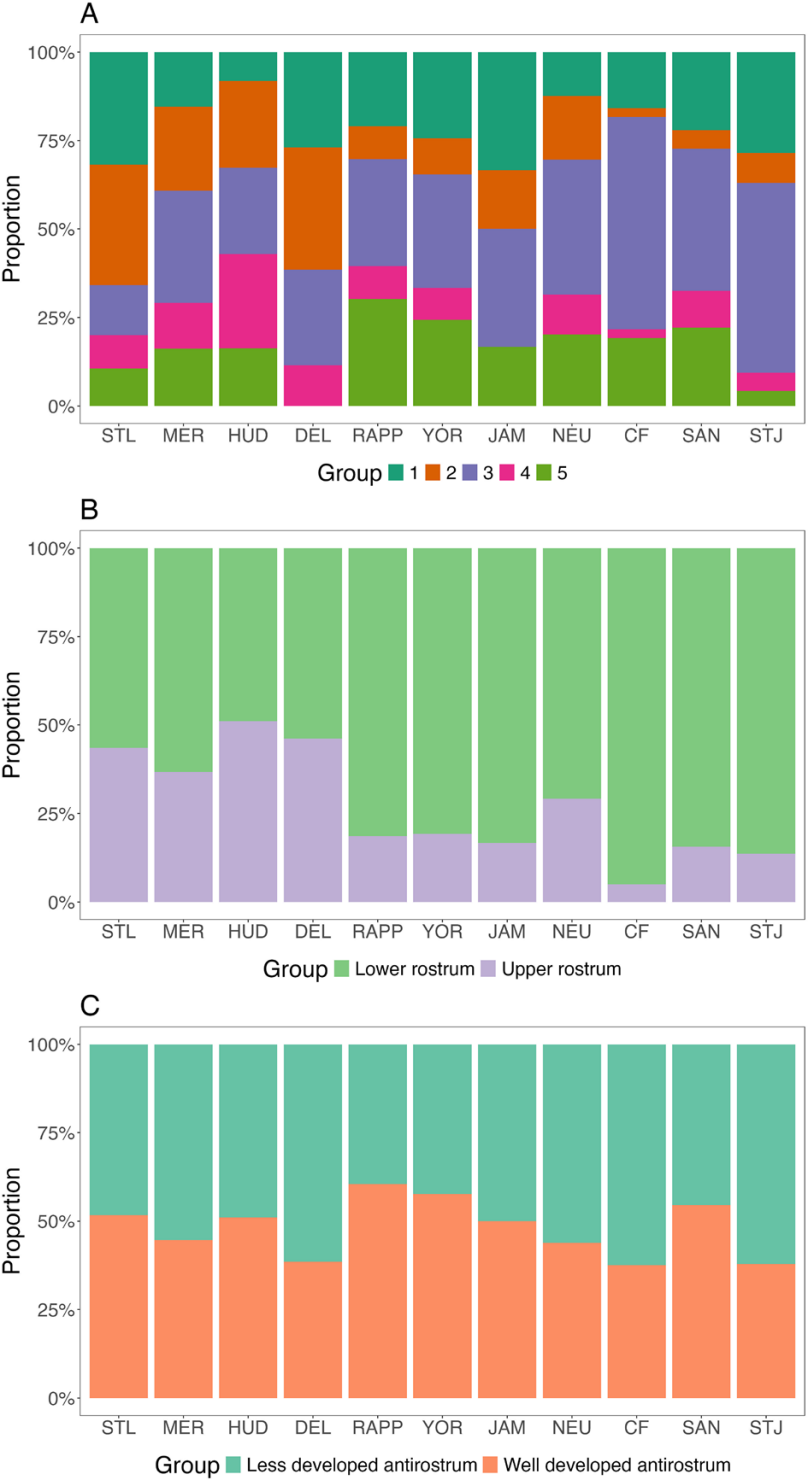
(**A**) Proportions of the five different morphotypes found in *A. sapidissima*, from eleven populations sampled across Quebec (QC) to Florida (FL). Sampling locations include the St. Lawrence (STL), Merrimack (MER), Hudson (HUD), Delaware (DEL), Rappahannock (RAPP), York (YOR), James (JAM), Neuse (NEU), Cape Fear (CF), Santee (SAN), and St. Johns (STJ) rivers. (**B**) Proportion of morphotypes showing a lower (M1, M3 and M5) or upper (M2 and M4) *rostrum*. (C) Proportion of morphotypes with less (M2 and M3) or well (M1, M4 and M5) developed *antirostrum*.

The proportions and distribution of morphotypes varied distinctly across rivers (Fig. 9A). M1, M2 and M3 were present in all rivers, with M2 and M3 exhibiting opposing latitudinal trends: M2 decreased while M3 increased from northern populations (St. Lawrence to Delaware) to southern populations (Rappahannock to St. Johns rivers). M5 was absent in the Delaware River, and M4 was absent in the James River, both of which had the smallest sample sizes among populations. Cape Fear showed the lowest proportions of M2 (2.5%) and M4 (2.5%) but the highest of M3 (60%), while the southernmost St. Johns River had the lowest proportion of M5 (4.3%) and a high proportion of M3 (53.6%).

When comparing morphotypes by *rostrum* morphology (Fig. 9B), northern populations (St. Lawrence to Delaware) exhibited a balanced proportion of upper *rostrum* (M2, M4) and lower *rostrum* (M1, M3, M5) otoliths, while southern populations (Rappahannock to St. Johns) were predominantly characterized by lower *rostrum* otoliths (81–95%).

Regarding *antirostrum* development (Fig. 9C), southern rivers such as Cape Fear (63%) and St. Johns (62%) showed the highest proportions of otoliths with a less developed *antirostrum* (M2, M3). In contrast, the Rappahannock (60%) and York (57%) rivers displayed the highest proportions of otoliths with a well-developed *antirostrum* (M1, M4, M5).

## Discussion

### Geographic and environmental drivers of otolith shape in American shad

American shad undertake long-distance migrations along the North American coast, as evidenced by tagging studies^40^, suggesting potential stock mixing at sea. Despite this, natal river fidelity predominates^41^, likely influenced by shorter northern spawning seasons that may limit straying^42^ and promote isolation by distance^43^. Human-mediated gene flow further complicates this structure, with extensive stocking programs since 1867 redistributing billions of shad among U.S. rivers^44,45^. Our findings revealed pronounced morphological heterogeneity in otolith contours across 11 rivers spanning Canada to Florida, enabling successful classification, particularly in the Delaware, York and James rivers, where classifications reached 100%, supporting the assumption of strict natal homing^46^. This heterogeneity is linked to five distinct morphotypes (M) present in differing proportions across the 11 rivers, which differ in *rostrum* and *antirostrum* morphology and size, as well as posterior margin patterns. While these differences enable classification, the extent of within-population variability suggests that multiple factors may shape otolith morphology beyond natal origin alone. Otolith development is influenced by both genetic and environmental factors, with certain features, such as *rostrum* and *antirostrum* size, likely having a stronger genetic basis, while the ventral contour is primarily shaped by environmental influences^47^. However, phenotypic plasticity may also play a role, as otolith shape can be modulated by exogenous factors such as habitat complexity, hydrodynamic conditions, and temperature regimes, which influence accretion patterns during early development^47–50^. The variation among morphotypes appears environmentally driven, consistent with Vignon^26^ observations of *Lutjanus kasmira*, showing that habitat shifts during ontogeny significantly altered otolith morphology. In shad, the first migration from natal rivers to the ocean may similarly shape otolith development, as early environmental exposures influence growth trajectories. For instance, Vignon^26^ demonstrated that environmental conditions can override genetic factors, reshaping the otolith morphology of bluestripe snapper (*Lutjanus kasmira*) during habitat transitions between channel and outer slope zones. Similarly, the distinct morphological differences between northern and southern American shad populations likely reflect environmental variability during early life stages. Southern populations, where morphotypes M1, M3, and M5 are more prevalent (e.g., Rappahannock to St. Johns rivers), exhibit otolith features similar to outer slope snappers, characterized by a lower *rostrum*. This trait may be associated with the warmer southern waters, where the annual mean SST exceeds $17\,^{\circ }$C, compared to the colder northern waters, with average below $15\,^{\circ }$C (Fig. 1). In contrast, northern populations (e.g., St. Lawrence to Delaware rivers), where morphotypes M2 and M4 dominate, exhibit otolith characteristics akin to channel snappers, including an elongated and upper *rostrum*. This upper *rostrum* morphology may not only be associated with lower mean SSTs but also with ultrasonic hearing. Species of the genus *Alosa* (Clupeiformes) can detect sounds up to 180 kHz—far beyond the 1–3 kHz range typical of most fish^51^. American shad, in particular, can perceive sounds up to at least 180 kHz^52,53^, responding strongly to pulses resembling dolphin echolocation signals. This advanced hearing capability likely evolved as an adaptation to detect echolocating dolphins their primary predators^53^, which are more abundant in northern waters, alongside other marine mammals. These northern regions, especially those north of Cape Hatteras, are characterized by high predator densities and distinctive bathymetric features such as continental shelf breaks, banks, and ledges^54^. Morphotypes M2 and M3, distinguished by a less developed *antirostrum*, align with enhanced swimming capacity^47^. This trait likely supports greater mobility in the semelparous southern populations (Cape Fear to St. Johns rivers). These populations, influenced by a prolonged evolutionary history in stable habitats persisting for hundreds of thousands of years, exhibit higher genetic diversity and phenotypic plasticity^46^. Extended spawning seasons in southern regions further contribute to increased gene flow and reduced isolation^46,55^. These ecological and evolutionary factors may explain the slightly lower classification accuracies (< 92%) observed in these populations, reflecting greater connectivity among rivers. However, despite the high mobility indicated by the presence of M2 morphotypes (35%) in the Delaware population, which suggests potential for high dispersal, strict natal homing behaviours were observed. This reflects the complexity of migratory strategies even within populations with morphotypes linked to increased mobility. In contrast, northern populations (e.g., St. Lawrence, Merrimack, and Hudson rivers) inhabit postglacial habitats formed approximately 10,000 years ago^46^. These populations experience shorter spawning seasons, which limit dispersal and reinforce isolation by distance^46,55^. The pronounced *antirostrum* development observed in northern morphotypes (M1, M4, and M5) may reflect adaptations that restrict mobility, favouring stronger site fidelity. For the hickory shad (*Alosa mediocris*), another anadromous species, significant differences in otolith shape were observed among 22 watersheds in Florida, Georgia, South Carolina, North Carolina, Virginia, Maryland, Delaware, and the District of Columbia, with most variation attributed to the regions of the *antirostrum*, *excisura ostii*, and *rostrum*^18^. Though not always apparent in the average shape of otolith wavelet coefficients, the prominence of the lower and upper *rostrum* suggests that these features are not exclusive to American shad (see^18^). Notably, similar *rostrum* characteristics have also been documented in *Alosa alosa* and *A. fallax*, further indicating that these traits may be widespread among anadromous clupeids^56^. This raises the question: Are these *rostrum* shapes specific to anadromous herrings, which must navigate complex riverine environments during their extensive migrations? Northern rivers along the U.S. East Coast are characterized by spring freshets—seasonal floods caused by melting snowpacks—which coincide with the spawning migrations of shad^57,58^. These high-flow conditions create turbulent environments that may necessitate specific adaptations in otolith morphology for balance and orientation. In contrast, southeastern rivers, generally more elongated below the fall line—a geomorphological break in topography with broad coastal plains leading to the sea^59^—are typically characterized by long stretches of slow-moving water, with extensive side channels, wetlands, and backwaters^60^. The distinct hydrodynamic regimes of these regions likely impose different selective pressures on the sensory systems of anadromous fish, potentially shaping otolith morphology to optimize navigation under varying flow conditions.

### Determining the number of distinguishable groups based on otolith morphology

The population dynamics of American shad are inherently complex, and this study reveals a nuanced structure that reflects this complexity. We identified at least five distinct groups based on otolith morphology: one in Canada (St. Lawrence River) and four in the U.S. (Merrimack + Hudson, Delaware, Rappahannock to Santee, and St. John’s). These findings are consistent with prior research by Hasselman et al.^46^, who, despite detecting weak genetic differentiation among U.S. populations, distinguished only two major clusters—one representing semelparous and the other iteroparous populations—out of the nine populations spanning from Canada to the U.S. Interestingly, their genetic evidence indicated three major barriers to gene flow, with a significant separation between the St. Lawrence River and all other populations. This genetic isolation is mirrored by our otolith morphology data, where the St. Lawrence population exhibited a unique contour shape, including a deeper *notch* and a pronounced *antirostrum*, reinforcing its distinctiveness. Canadian populations, which have been largely unaltered by stock transfers, likely retain the natural patterns of gene flow and genetic drift that have developed since postglacial colonization^46^. This natural isolation, paired with the morphological and geochemical evidence, underscores the distinctiveness of the Canadian shad populations. In addition to morphological differentiation, otolith microchemistry further supports the existence of these groups. Previous studies have revealed high Sr:Ca ratios in otoliths from southern rivers, particularly the St. John’s, indicative of the distinct geology and geochemistry in these regions^11,13^. Similarly, Delaware juveniles have been characterized by unique elemental ratios (e.g., Mg:Ca, Mn:Ca, Sr:Ca, and Ba:Ca), further distinguishing them from other populations^11^. These chemical signatures offer additional evidence of regional structure and variability in otolith composition. Otolith contour analysis confirmed the broader groupings identified by genetic and chemical studies, while also revealing finer-scale structure—distinguishing five groups, including four within the U.S. Unlike genetics or microchemistry, it captures phenotypic variation shaped by development and ecology, allowing detection of subtle spatial differences among populations.

## Conclusion

Metapopulation dynamics, particularly straying rates, provide additional insight into the observed patterns. Straying, a characteristic feature of metapopulations, may buffer against environmental disturbances and habitat fragmentation^43^. Our study, showing misclassification rates ranging from 5% (Rappahannock) to 18% (St. Johns), suggests that actual straying rates may exceed the 3% commonly assumed for American shad^61^. While this could reflect true straying events, some level of misclassification may also stem from differences in alosid juvenile habitat use rather than movement between populations. Research has shown that the availability and extent of suitable nursery habitats influence how long juveniles remain in freshwater before marine migration, with some populations exhibiting delayed emigration when more habitat is accessible^62^. Additionally, studies on blueback herring (*Alosa aestivalis*) in the Hudson River watershed indicate that many juveniles remain in nursery areas beyond their first year, re-entering them after winter for additional growth^63^. These alternative processes may contribute to the observed misclassification rates, highlighting the complexity of interpreting straying solely through otolith-based classification. Nevertheless, habitat fragmentation and historical stock transfers likely play a role in the observed connectivity patterns, with environmental factors in neighboring rivers facilitating gene flow^64,65^. These findings align with the model proposed by Poulet et al.^66^, which emphasizes the role of straying in shaping population structure, particularly under anthropogenic pressures and environmental fluctuations.

To conclude, we found significant variation in otoliths of different American shad populations. However, our sampling of rivers was based upon what was available, and missed many rivers where shad are known to spawn. Future research could fill in gaps throughout the range and might target systems where hypotheses about form and function could be tested.

## Supplementary Information

Supplementary Information.

## Data Availability

The data that support the findings of this study are available from the corresponding author upon reasonable request.
