# Supplementary material for: Phenotypic variation in otolith shape of American shad across eastern North American rivers
Source: Sci Rep. 2025 Aug 13;15:29748. doi: 10.1038/s41598-025-14742-1 (PMC12350608; doi:10.1038/s41598-025-14742-1)
Supplement: Supplementary file 1 — Supplementary Information. [file 41598_2025_14742_MOESM1_ESM.pdf]

1 SUPPLEMENTARY TABLES AND FIGURES

1.1 Tables

Table S1. Post-hoc pairwise comparison results with Bonferroni corrections for total variation across population.

River	STL	MER	HUD	DEL	RAPP	YOR	JAM	NEU	CF	SAN
MER	0.055									
HUD	0.055	1.000								
DEL	0.990	1.000	0.220							
RAPP	0.055	0.055	1.000	0.055						
YOR	0.055	0.165	1.000	0.055	1.000					
JAM	0.055	1.000	1.000	0.495	1.000	1.000				
NEU	0.055	1.000	1.000	0.055	1.000	1.000	1.000			
CF	0.055	0.055	0.055	0.055	0.275	0.055	1.000	0.055		
SAN	0.055	0.055	1.000	0.055	1.000	1.000	1.000	1.000	0.110	
STJ	0.055	0.055	0.055	0.055	0.055	0.055	1.000	0.055	0.055	0.055

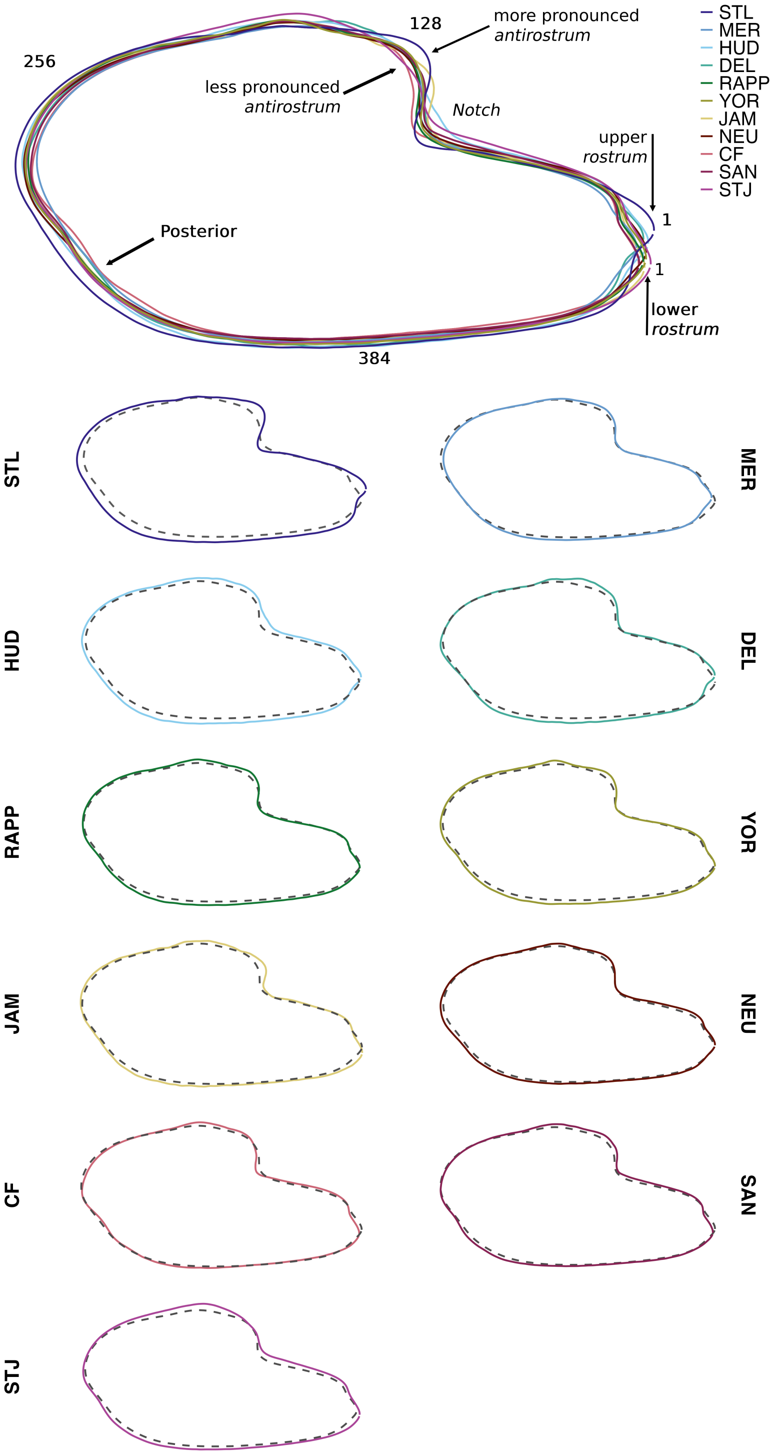

Figure S1. Mean reconstructed otolith shapes representing the average phenotype of *A. sapidissima* from eleven populations sampled across Quebec (QC) to Florida (FL). Sampling locations, listed from north to south, include the St. Lawrence (STL), Merrimack (MER), Hudson (HUD), Delaware (DEL), Rappahannock (RAPP), York (YOR), James (JAM), Neuse (NEU), Cape Fear (CF), Santee (SAN), and St. Johns (STJ) rivers. The dashed line represents the overall mean otolith contour across all populations combined. The first Cartesian coordinate is automatically determined by selecting the farthest point from the centroid to the otolith outline.

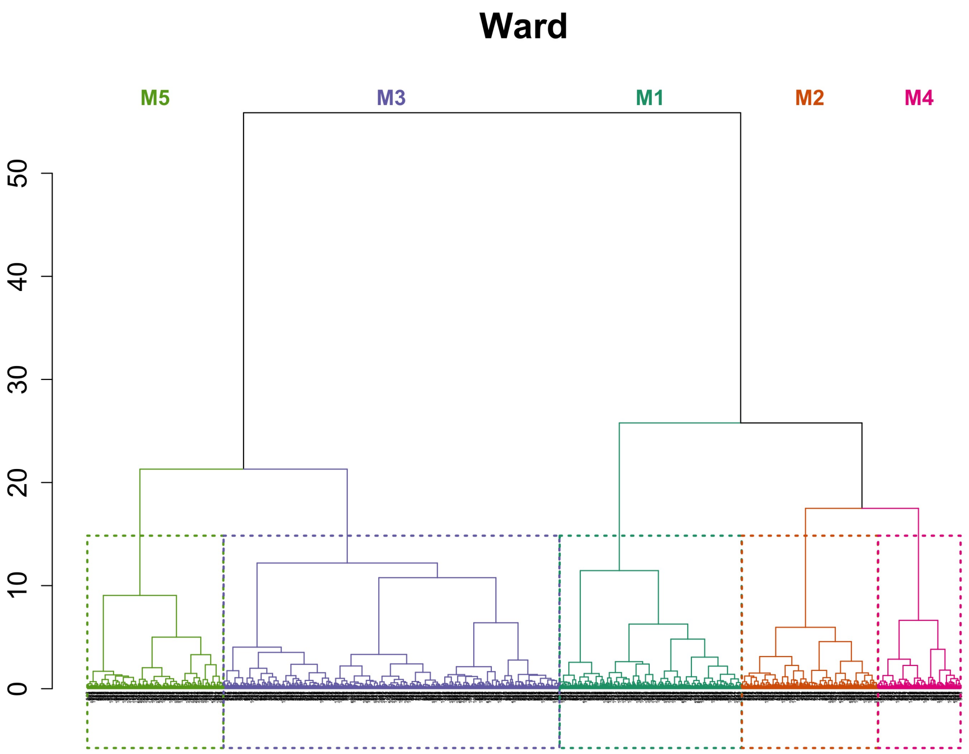

Figure S2. Hierarchical cluster analysis dendrogram generated from a distance matrix computed using principal component analysis results. The dendrogram illustrates clustering outcomes obtained with the Ward method for *A. sapidissima*, samples collected from eleven populations sampled across Quebec (QC) to Florida (FL). Sampling locations include the St. Lawrence (STL), Merrimack (MER), Hudson (HUD), Delaware (DEL), Rappahannock (RAPP), York (YOR), James (JAM), Neuse (NEU), Cape Fear (CF), Santee (SAN), and St. Johns (STJ) rivers.
